# The modern treatment of the simple bone cysts

**Published:** 2012-12-25

**Authors:** A Ulici, R Balanescu, L Topor, M Barbu

**Affiliations:** * “Carol Davila" University of Medicine and Pharmacy, Bucharest, Romania; ** Department of Pediatric Surgery and Orthopedics “Grigore Alexandrescu" Clinical Emergency Hospital for Children, Bucharest

## Abstract

This study was performed between 2007-2012 and encompasses 94 patients. The patients were divided in two groups. The first group included the patients who have benefited from surgical treatment (42 cases) and the second one included patients who benefited from conservative treatment. Out of the total number of cases, 63 cases showed an intact simple bone cyst that was most of the time an accidental discovery. 31 patients presented with fracture sustained on a simple bone cyst. There were 63 boys and 31 girls. Their mean age was 9.9 +/- 2.34 years. Single injection was performed for 49 patients; the rest had double or triple injections. The mean follow-up was 34.5 +/- 6.6 months. The procedure succeeded in obtaining healing in 77 cysts (82%). Cyst index of more than five and cortical index of less than 1 mm were significantly prone to pathological fractures and had significant poor results after treatment. Our results suggested that an autogenous bone marrow injection is a safe and effective treatment method for simple bone cysts, when compared with the surgical management, but sometimes-repeated injections are necessary. Cyst index and cortical width are good indicators for treatment outcome.

## Introduction

The simple bone cyst is the most common benign lytic bone lesion in childhood, mainly affecting the proximal femur and proximal humerus. The simple bone cyst (SBC) also called unicameral bone cyst is a tumor-like lesion of unknown cause, attributed to a local disturbance of the bone growth [**3,6,9,17**]. Although the pathogenesis is still unknown [**10,11**], the lesion appears to be reactive or developmental rather than to represent a true neoplasm [**1**]. SBC consists of a solitary cavity lined by a membrane of variable thickness and filled with a clear yellow fluid [**2**]. It represents approximately 3% of all primary bone lesions [**4**].

 Until now, only two SBC’s have been examined cytogenetically, revealing complex clonal rearrangements involving chromosomes 4,6.8,12,16 and 21 in one case and a translocation (p11.2; q13) in the second case [**14,15**]. In this case, one examination of the fourth recurrence by polymerase chain reaction revealed a TP53 mutation [**16**].

 The SBC is more common in males 3:1 and is usually detected during the first 2 decades of life [**1,13**]. About 65% of these cysts occur in teenagers and an additional 20% in the first decade of life. The vast majority of SBC are located in the proximal diaphysis of the humerus and femur, especially when they occur in patients younger than 17 years old [**9,12**]. The symptoms include pain, swelling, or stiffness at the nearest joint. (3) A pathologic fracture is often the first sign of the lesion. (6,9) In fact, this is the most common complication of the SBC and occurs in about 66% of the cases. In older patients the incidence of involvement of atypical sites, such as calcaneus, talus, and ilium rises significantly [**8,13**]. In these sites, the lesion is usually asymptomatic and is discovered by accident.

**Fig. 1:**
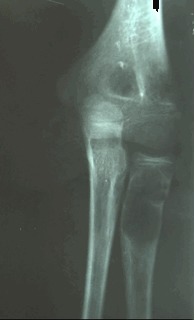
Radial bone cyst

 Radiographically, the appearance of an SBC is that of a centrally located, well-circumscribed, radiolucent lesion with sclerotic margins. The lesion is located within the diaphysis or the metaphysis of a long bone, abutting or being remote from the cartilaginous growth plate (**Fig. 1**). Epiphyseal extension is unusual [**5,8**].

 The cortex is frequently thinned and cortical expansion may be present, but the width of the SBC does not exceed the width of the neighboring plate [**7**]. A periosteal reaction is absent unless there has been a pathologic fracture. Diagnosis is best based on conventional radiographs; conventional tomography and CT are used only exceptionally in equivocal cases.

 The aim of treating SBC is to prevent all possible complications and to avoid prolonged restriction of physical activity. The appropriate treatment, however, remains uncertain and a wide spectrum of treatment modalities have been proposed ranging from observation to subtotal resection. 

 Bone grafting has been disappointing because of the recurrence rate and considerable morbidity. Percutaneous injection of steroids was introduced first by Scaglietti et al. who reported a success rate of up to 90%. The low morbidity and simplicity of this treatment explains its popularity. More recently, bone marrow has been proposed as an alternative to steroids because of osteoinductive potential. The purpose of this study was to evaluate the clinical outcome of treating SBC’s by aspiration and percutaneous auotogenous bone marrow injection (ABMI). The effectiveness of this method was studied according to different factors that affect the outcome including the aggressiveness of the cyst.

## Materials and methods

**Fig. 2:**
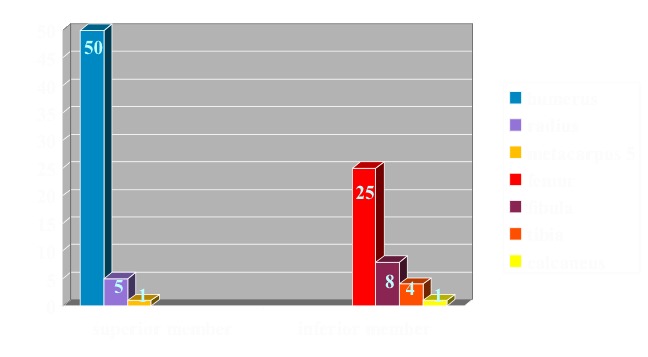
Case distribution according to their disposal

From 2007 to 2012, 94 consecutive patients with diagnosis of SBC were treated in the authors’ institute by: aspiration and percutaneous ABMI or steroid injection (methylprednisolone), and surgical treatment. Confirmation of the diagnosis was based on characteristic radiographic appearance and histological study of the specimen taken by aspiration. There were 63 boys and 31 girls. The mean age at the outset of treatment was 9.9 +/- 2.34 years (range 6-17). The cyst localizations were humerus 50 cases, radius 5 cases, V-th metacarpal 1, femur 25, peroneus 8, tibia 4, calcaneus 1 (**Fig. 2**). 

**Fig. 3:**
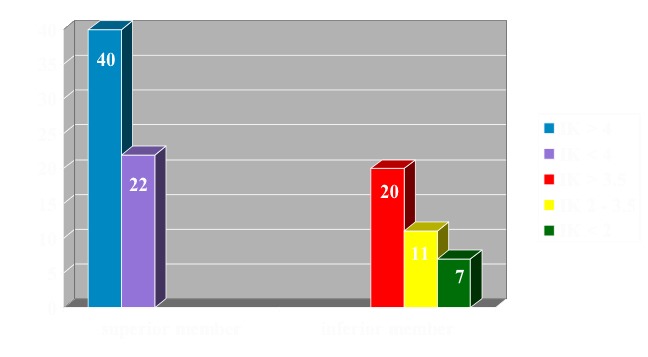
Case distribution according to the cystic index

55 cysts were uniloculated and 39 were multiloculated. Cyst size is defined as the ratio of the length of the cyst to the width of the adjacent physis, which allows a direct comparison of the sizes without requiring correction factors for different ages. The mean size of all cysts in this study was 1.857 +/- 0.473 (range 0.9-3.1). The fracture risk was evaluated by measuring the cyst index of Kaelin and Mc Ewan and the cortical thickness. The cyst index is the cyst area divided by the squared diaphyseal diameter. One or more trapezoids are drawn around the cyst area. Mechanically, a cyst is considered not prone to fracture when the index is lower than 3 and the cortical width is lower than 2 mm. The patients were divided by Cyst Index (CI) as it follows: upper limb with a CI >4 – 40 patients, upper limb with a CI <4 – 22 patients, lower limb with a CI <2 – 7 patients, lower limb with a CI 2-3,5 – 11 patients, lower limb with a CI > 3,5 – 20 patients (**Fig. 3**).

**Fig. 4:**
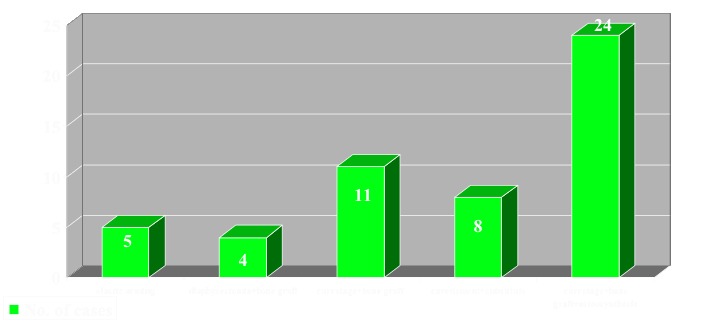
Case distribution according to the applied surgical treatment

Surgical treatment consisted in elastic reinforcement 5 cases, dyaphisectomia + bone graft 4 cases, curettage and bone grafting 11 cases, curettage of cystic cavity and osseous substitute 8 cases, curettage of the cystic cavity and structuralized bone graft plus stabilization 24 cases (**Fig. 4**).

Conservative treatment was represented by cast immobilization 15 cases, steroid injection 35 cases, and ABMI 12 cases. Simple cast immobilization was applied to those cases of SBC who sustained a pathologic fracture without fragment displacement. ABMI and steroid injections were performed in cases of intact cysts or after the occurrence of fracture healing. The technique of ABMI and steroid injection are identical, the only difference being the kind of material used for the injection. In our institution, we employed the following technique of ABMI:

-with the patient under total anesthesia, we visualize the cystic cavity with the help of fluoroscopic guidance.

-several bone needles are introduced in the proximal and the distal pole of the cystic cavity.

-the cyst cavity was evacuated without force and the fluid was sent to histopathological examination.

- the cavity was thoroughly flushed with normal saline.

-cystogram with contrast substance was performed to determine if the cyst was uniloculated or multiloculated.

-the bone marrow was harvested from the iliac crest using a bone marrow aspiration needle.

The presence of LDH was highlighted within the liquid extracted from the cystic cavity, which was 2-3 times greater than the ordinary values found in blood; also, the alkaline phosphatase had an increased level of more than 10 times then the average serum level.

## Results

**Fig. 5:**
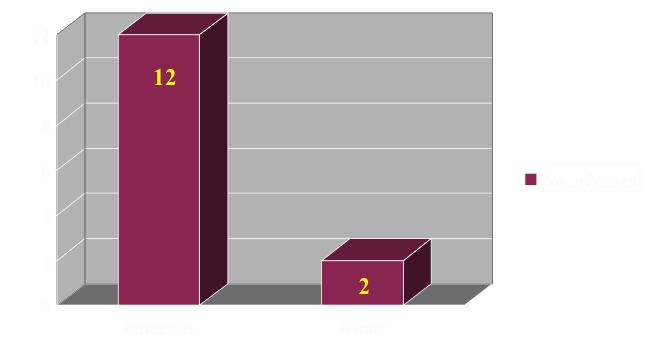
Case distribution according to post-surgical treatment recurrences

 In the quantification of the results, we considered the occurrence of the recidivated cysts, time span until the total healing and the number of injections needed in the case of steroid injection and ABMI. In the case of surgical management, we encountered recidivated cysts in 14 patients. Upper limb accounted for 12 recidivated cysts and the lower limb for only 2 (**Fig. 5**).

**Fig. 6:**
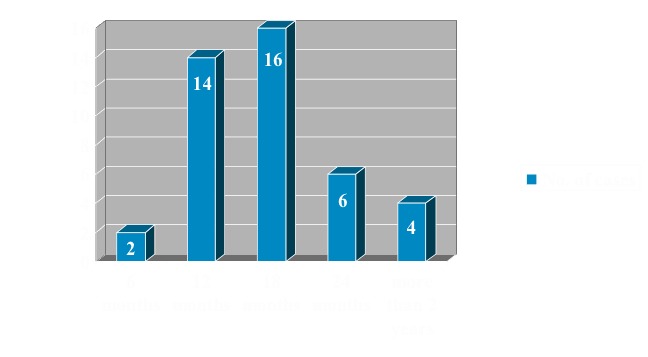
Case distribution according to post-surgical treatment recurrences

 Time span from the surgical intervention to total healing of the cyst varied between 6 months and 2 years with a peek incidence between 12 and 18 months (30 patients) (**Fig. 6**).

 It was observed that precocious healing (before age 1 from the beginning of the treatment) occurred in the cases with latent cysts, and for the cases which needed prolonged periods of time for healing several surgical procedures were needed.

**Fig. 7:**
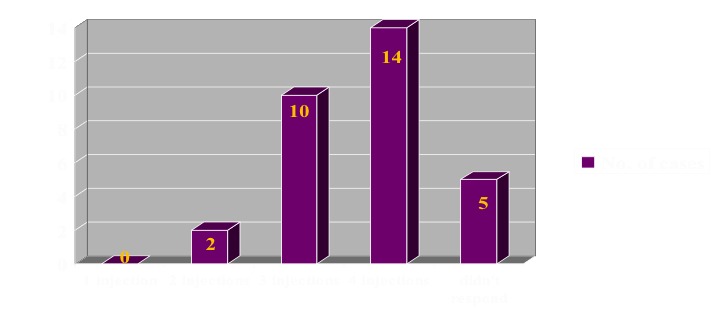
Methylprednisolone injection case repartition according to the required injections number

 From the conservative point of view, in the case of steroid injection, the quantification of the results was also based on the number of injections to achieve healing. According to the number of injections until healing, patients were distributed as it follows 1 injection -0 cases, 2 injections- 2 cases, 3 injections 10 cases, 4 injections – 14 cases. Five patients had no response to the treatment whatsoever, and they were managed surgically (**Fig. 7**). 

We used ABMI to 12 of our patients. The first and most important conclusion was that most of the patients (9) had a good response since their first injection. According to case partition and taking into account the time span from the debut of the conservative treatment and the mechanical healing of the cyst (CI lower than 3 and cortical width lower than 2 mm), we observed that most of the patients were grouped in the 6 months-1 year interval. The cases that needed more time for the healing were most of them part of the cast immobilization group. 

## Discussion

After the surgical management, a large proportion of the patients sustained a recidivated cyst. These recidivated cysts were encountered in those cases that were treated with curettage and osseous graft or osseous substitute (**Fig. 8a, Fig. 8b**).

**Fig. 8a:**
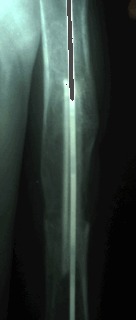
Humerus bone cyst – 1 month after surgical treatment

**Fig. 8b:**
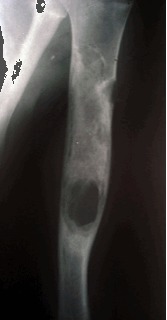
Humerus bone cyst – 15 months after surgical treatment

After a brief period of time that mimed healing, we encountered the reappearance of the cyst, most of the time in the inferior pole of the old cyst. In the upper limb most of the recidivated cyst appeared in the humerus. The only surgical management that did not lead to a recidivated cyst was elastic reinforcement and osseous reconstruction with diaphysectomy. The only impediment related to simple elastic reinforcement was the period of time needed for the healing of the lesion, which proved to be very long. In the case of osseous reconstruction with diaphysectomy, this procedure is prone to a far greater morbidity rate. We recommend this kind of treatments only in the cases of conservative management failure and in cases of displaced pathological fracture. The conservative treatment consisted in simple cast immobilization of the pathological fracture localized in the upper limb and without fragment displacement, ABMI or steroid injections. The last two methods of treatment were employed only in those cases that sustained a cyst without a pathological fracture or after the healing of a previous pathological fracture, which did not lead to cyst healing. Those cases that sustained a pathological fracture localized at the level of the upper limb were successfully treated by conservative means, the result being the healing of the fracture and subsequently of the cyst. In the case of the steroid injection, we encountered 5 cases that had no response, in comparison with ABMI, in which case we encountered a 100% rate of lesion healing, even from the first injection (**Fig. 9a, Fig. 9b**).

**Fig. 9a:**
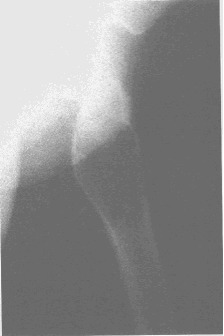
Femoral bone cyst – injection’s time

**Fig. 9b:**
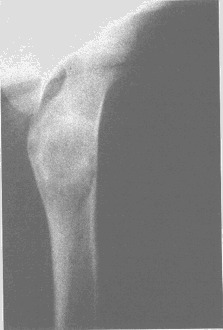
Femoral bone cyst – 3 months after injection

## Conclusion

- A recidivated cyst frequently follows surgical management by the method of curettage and grafting.

 - Diaphysectomy and osseous graft leads to a hundred percent healing, but it is followed by great rates of morbidity and it is unnecessarily laborious.

 - The logical treatment for SBC, that sustained a pathological fracture, as well as in the case of an ordinary fracture. 

 - Steroid injection may be accompanied by failure, and only exceptionally, healing occurs before the third injection.

 - ABMI is a simple cost free method, effective in both active cyst and inactive cyst, and certain signs of healing follow it in most of the cases since the first injection.
